# A Novel Ideal Radionuclide Imaging System for Non-invasively Cell Monitoring built on Baculovirus Backbone by Introducing Sleeping Beauty Transposon

**DOI:** 10.1038/srep43879

**Published:** 2017-03-06

**Authors:** Jing Lv, Yu Pan, Huijun Ju, Jinxin Zhou, Dengfeng Cheng, Hongcheng Shi, Yifan Zhang

**Affiliations:** 1 Department of Nuclear Medicine, Ruijin Hospital, Shanghai Jiao Tong University School of Medicine, Shanghai, China; 2 Department of Nuclear Medicine, Zhongshan Hospital, Fudan University School of Medicine, Shanghai, China

## Abstract

Sleeping Beauty (SB) transposon is an attractive tool in stable transgene integration both *in vitro* and *in vivo*; and we introduced SB transposon into recombinant sodium-iodide symporter baculovirus system (Bac-NIS system) to facilitate long-term expression of recombinant sodium-iodide symporter. In our study, two hybrid baculovirus systems (Bac-eGFP-SB-NeoR and Bac-NIS-SB-NeoR) were successfully constructed and used to infect U87 glioma cells. After G418 selection screening, the Bac-eGFP-SB-NeoR-U87 cells remained eGFP positive, at the 18^th^ and 196^th^ day post transfection (96.03 ± 0.21% and 97.43 ± 0.81%), while eGFP positive population declined significantly at 18 days in cells transfected with unmodified baculovirus construct. NIS gene expression by Bac-NIS-SB-NeoR-U87 cells was also maintained for 28 weeks as determined by radioiodine uptake assay, reverse transcription-polymerase chain reaction (RT-PCR) and Western Blot (WB) assay. When transplanted in mice, Bac-NIS-SB-NeoR-U87 cells also expressed NIS gene stably as monitored by SPECT imaging for 43 days until the tumor-bearing mice were sacrificed. Herein, we showed that incorporation of SB in Bac-NIS system (hybrid Bac-NIS-SB-NeoR) can achieve a long-term transgene expression and can improve radionuclide imaging in cell tracking and monitoring *in vivo*.

The radionuclide reporter gene systems register their superiority over other reporter gene systems on sensitivity and specificity. The sodium-iodide symporter (NIS), a plasma membrane protein which mediates the active accumulation of I^−^ from the interstitium into the cell by the inwardly directed Na+ gradient^1^,^2^, is well known as a non-immunogenic, non-invasive, and non-complex probe synthesis reporter gene, which can be visualized by SPECT or PET in real-time monitoring gene expression, stem cells, and immune cells in pre-clinical and clinical models^3^,^4^,^5^,^6^.

Despite NIS being an ideal reporter gene for non-invasive imaging in molecular imaging, the delivery system is still the crucial process before the NIS reporter gene can be visualized *in vivo*. Non virus vector^7^ and viral vectors, such as adenovirus vector^8^,^9^, adeno-associated virus vector^10^, retrovirus vector^4^,^11^, and lentivirus vector^3^,^12^ were used for transferring NIS gene into cells both *in vitro* and *in vivo*. However, adverse immune reaction, insufficient packaging capacity, manufacturing and storing difficulties, as well as safety concerns of insertional mutagenesis^13^, limit the application of these vectors in NIS gene delivery. With better biosafety, large cloning capacity, low cytotoxicity, as well as easy manipulation and production^14^, the baculovirus delivery system mediated NIS gene expression had shown its advantages in non-invasive imaging *in vivo* in our previous study^5^,^15^,^16^,^17^.

However, the baculovirus mediated transgenes do not integrate into chromosomes^15^,^17^,^18^, and only have transient transgene expression in less than two weeks. Thus making long-term expression of NIS reporter gene in a recombinant baculovirus system is crucial for the further improvement of Bac-NIS system in non-invasive molecular imaging *in vivo*.

Transposon system, also known as mobile genetic elements, is a non-viral, DNA-mediated gene transfer system, which can efficiently insert genetic information into host chromosomes^18^,^19^. The “sleeping beauty” transposon (SB), originally isolated from Salmonid fish, is a member of Tc1/mariner superfamily of DNA transposon and is reactivated by molecular reconstruction of the elimination of the inactivating mutations in 1997^20^. It consists of two components: a transposon and a transposase which recognizes the inverted repeat containing direct repeated sequence (IR/DR) flanking the transgene in a transposon^19^,^21^,^22^. Until now, SB has been proven as an attractive tool of efficient and stable transgene integration both *in vitro* and *in vivo*^23^,^24^,^25^.

SB100X, one of the hyperactive versions of SB transposases, possesses a 100-fold more active, as well as a higher integration efficiency, into the host genome than the original version^26^. Thus, to cross the barrier of transient gene expression of the Bac-NIS system, we introduced SB100X into the backbone of recombinant baculovirus vector to obtain a novel hybrid system, which combined the advantages of these two systems. To better improve the novel system, the neomycin/geneticin resistance gene (NeoR) was also introduced into the hybrid system (Bac-NIS-SB-NeoR).

We herein evaluated the long-term reporter gene expression mediated by this novel constructed hybrid system and explored the potential application of this hybrid system in cell tracking and monitoring *in vivo*.

## Results

### The virus production of Bac-eGFP-SB-NeoR and Bac-NIS-SB-NeoR

**Figure 1:**
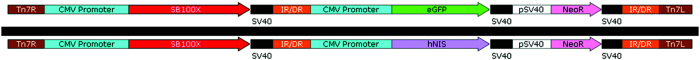
The schematic of constructed plasmids. The eGFP and NIS gene both driven by a CMV promoter were inserted into two IR/DR sites whereas SB100X driven by a CMV promoter was outside the two IR/DR sites in a single plasmid. CMV promoter = cytomegalovirus promoter, IR/DR = inverted repeat/direct repeated sequence, SB100X = Sleeping Beauty 100X.

The plasmids pFast-eGFP-SB-NeoR and pFast-NIS-SB-NeoR (Fig. 1) were constructed, and recombinant baculovirus were produced. The titers of Bac-eGFP-SB-NeoR and Bac-NIS-SB-NeoR were (8.68 ± 0.18) * 10^8^ and (4.01 ± 0.27) * 10^8^, respectively.

### Establishment of stable cell lines

**Figure 2:**
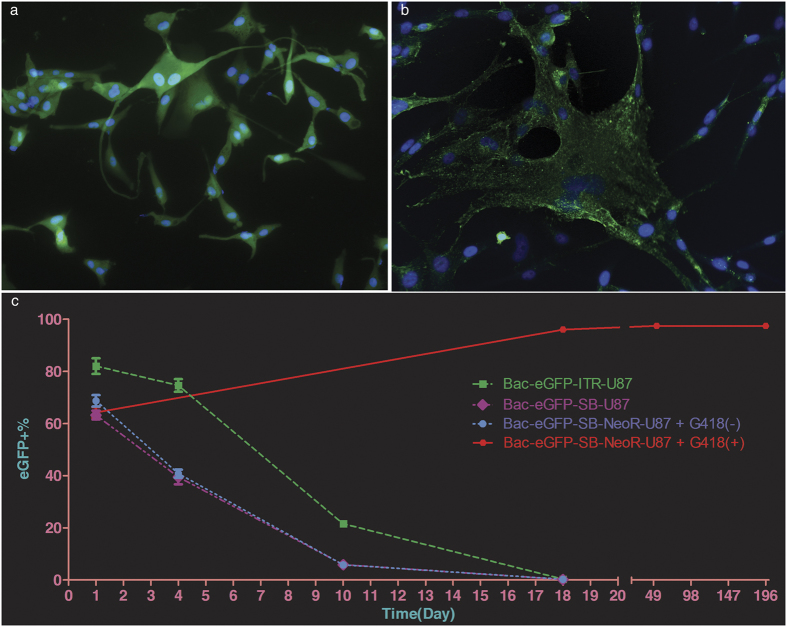
The long-term eGFP and NIS gene expression mediated by the novel hybrid system. The positive proportion of transgene expression at the 196^th^ day after G418 screening in Bac-eGFP-SB-NeoR-U87 (**a**) and Bac-NIS-SB-NeoR-U87 (**b**) cells was observed by fluorescence microscope. No significant decline (one-way ANOVA, p = 0.41) of eGFP+% from the 18^th^ day to the 196^th^ day in G418 screened Bac-eGFP-SB-NeoR-U87 cells was determined by flow cytometry (**c**), whereas a significant decline (one-way ANOVA, p < 0.0001) in other baculovirus infected U87 cells (**c**). eGFP+% = the percentage of eGFP positive cells, Data = means ± SD (at least N = 3).

After G418 screening, the majority of the Bac-eGFP-SB-NeoR-U87 cells expressed eGFP protein was confirmed by fluorescence microscope at the 196^th^ day (Fig. 2a) and flow cytometry at the 1^st^, 18^th^, 53^th^ and 196^th^ day post infection (Fig. 2c), which was 64.27 ± 3.64%, 96.03 ± 0.21%, 97.50 ± 1.00% and 97.43 ± 0.81%, respectively. No obvious decline of eGFP expression in G418 screened Bac-eGFP-SB-NeoR-U87 cells was observed between 18^th^ and 196^th^ day, whereas a significant decline of eGFP expression in Bac-eGFP-ITR-U87 (original version), Bac-eGFP-SB-U87 (with no screening resistance gene) and Bac-eGFP-SB-NeoR-U87 (with no G418 screening) cells in less than 18 days (Fig. 2c). Meanwhile, the majority of the Bac-NIS-SB-NeoR-U87 cells expressed NIS protein at the 196^th^ day was confirmed by cell immunofluorescence (Fig. 2b).

### Function determination of NIS protein expressed by Bac-NIS-SB-NeoR-U87 cells

**Figure 3:**
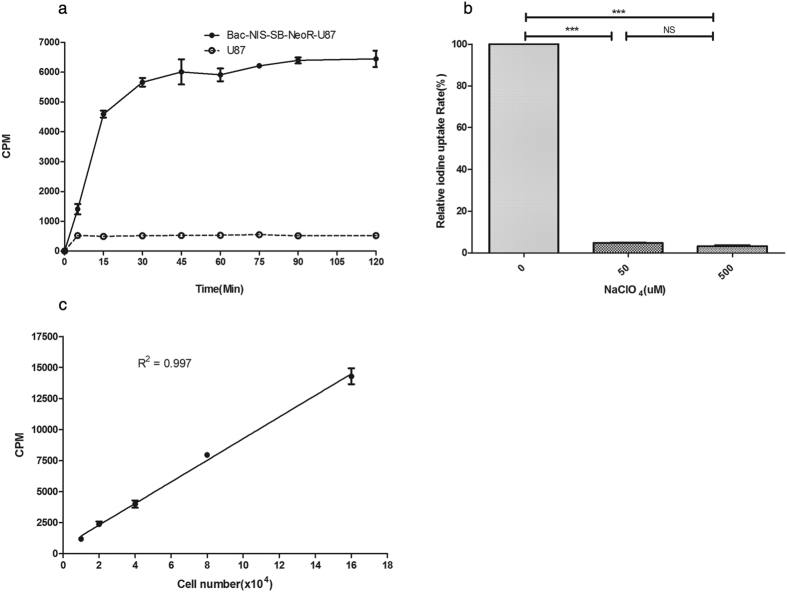
The specific function assay of NIS protein in screened Bac-NIS-SB-NeoR-U87 cells. The dynamic iodine uptake curve in screened Bac-NIS-SB-NeoR-U87 cells (**a**) and significant (two tailed Student’s t-test, p < 0.0001) inhibition effects by NaClO_4_ at various concentrations (**b**). A significant (p < 0.0001, R^2^ = 0.997) correlation between radioactivity and cell number of screened Bac-NIS-SB-NeoR-U87 cells (**c**). “***” = p < 0.0001, NS = no statistically significant difference. Data = means ± SD (at least N = 3).

In dynamic iodide uptake assay (Fig. 3a), the uptake of ^125^I in Bac-NIS-SB-NeoR-U87 cells dramatically increased and reached its peak at 45 minutes with no significant decease till 120 minutes. Two independent experiments with triplicates showed consistent results.

In the iodide uptake inhibition assay (Fig. 3b), the radioactivity uptake in Bac-NIS-SB-NeoR-U87 cells was significantly inhibited by various concentrations of NaClO_4_.

### Correlation analysis between radioactivity and number of Bac-NIS-SB-NeoR-U87 cells *in vitro*

The correlation analysis between the radioactivity of iodide uptake and the number of Bac-NIS-SB-NeoR-U87 cells displayed a high correlation (R^2^ = 0.997, p < 0.0001), which provided a potential invasive and quantitative method in evaluating the viable cell number by determining the intracellular radioactivity (Fig. 3c).

### Long-term expression of NIS in Bac-NIS-SB-NeoR-U87 cells

**Figure 4:**
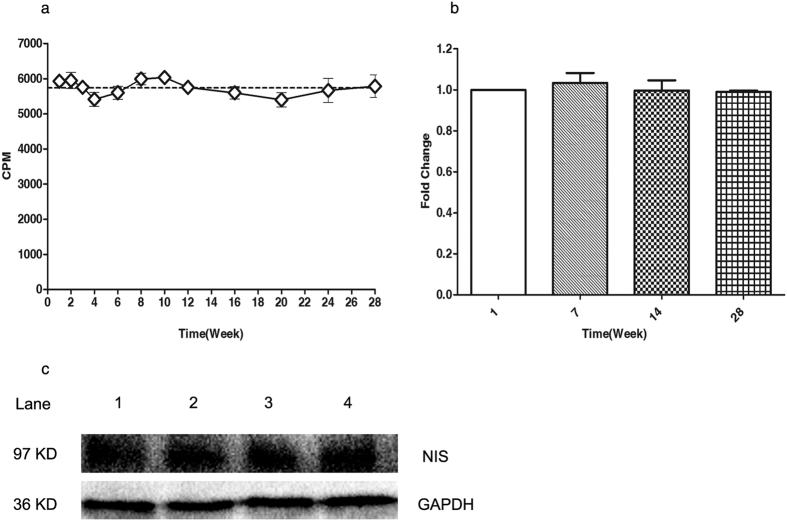
The long-term NIS expression assay. No significant (one-way ANOVA, p = 0.81) decline of NIS gene expression in screened Bac-NIS-SB-NeoR-U87 cells was determined by radioiodide uptake (**a**), mRNA level (**b**) and protein level (**c**) in various weeks. Lane 1, 2, 3, and 4 means 1^st^, 7^th^, 14^th^, and 28^th^ week. Data = means ± SD (at least N = 3).

There was no significant change of iodide uptake by Bac-NIS-SB-NeoR-U87 cells for 28 weeks under G418 selection screening (Fig. 4a). The NIS mRNA expression level was maintained through 28 weeks (Fig. 4b) and there was no decrease of NIS protein either as shown by western blotting analysis (Fig. 4c).

### ^131^I imaging *in vivo* by SPECT with pinhole detector

**Figure 5:**
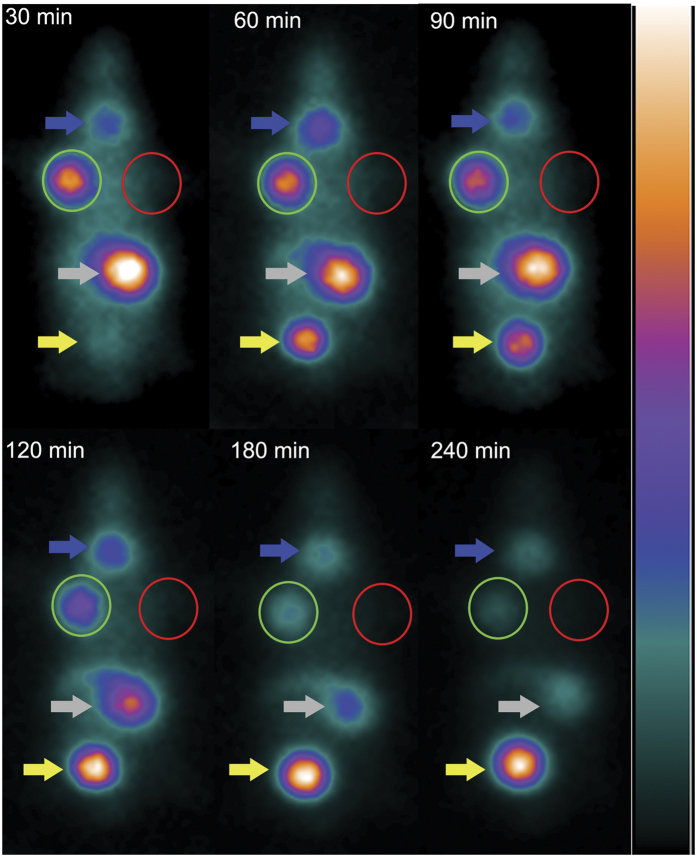
The dynamic imaging by SPECT instrument. The imaging variation over time in the first SPECT imaging (20^th^ day after transplantation with screened Bac-NIS-SB-NeoR-U87 cells). The green and red circle respectively represent the Bac-NIS-SB-NeoR-U87 cells and U87 cells. Blue, gray and yellow arrows respectively represent thyroid, stomach and bladder.

**Figure 6:**
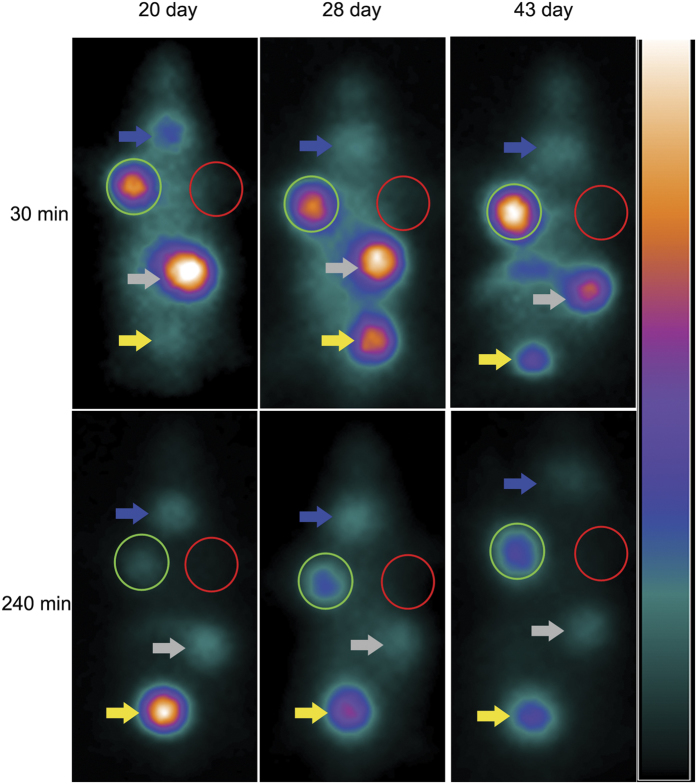
Three imaging at different days after transplantation with screened Bac-NIS-SB-NeoR-U87 cells by SPECT. The successfully detecting of Bac-NIS-SB-NeoR-U87 tumor cells at 30 minutes in each SPECT imaging *in vivo*. The green and red circle respectively represent the Bac-NIS-SB-NeoR-U87 cells and U87 cells. Blue, gray and yellow arrows respectively represent thyroid, stomach and bladder.

**Figure 7:**
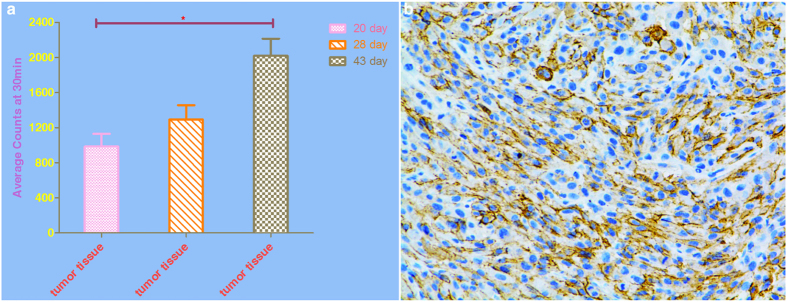
The analysis of radioactivity uptake and immunohistochemistry in tumor tissue. The average radioactivity uptake at 30 minutes corresponding to SPECT images (**a**) and the abundant NIS protein detected by immunohistochemistry (**b**) in Bac-NIS-SB-NeoR-U87 tumor tissue were performed. “*” = p < 0.05 (one-way ANOVA), Data = means ± SD (at least N = 3).

With a pinhole detector, the SPECT imaging at 30 minutes post tail vein injection of ^131^I clearly revealed the tumor in the right armpit, which representing Bac-NIS-SB-NeoR-U87 cells transplanted 20 days earlier. However, no significant signal was detected in the left armpit, where U87 cells were transplanted. The tumor image faded gradually from 60 minutes through 240 minutes post injection (Fig. 5). At 28 days and 43 days post transplantation, the tumor formed Bac-NIS-SB-NeoR-U87 cells was still visible at 30 minutes post injection (Fig. 6). In addition, the average radioactivity uptake in tumor tissue at 30 minutes in the SPECT images was also presented (Fig. 7a).

### Expression of NIS protein in tumor tissue of nude mice

The results of immunohistochemistry revealed abundant expression of NIS protein in the tumor tissue, mostly on the membrane of Bac-NIS-SB-NeoR-U87 cells (Fig. 7b).

## Discussion

Since Bac-NIS system cannot integrate target gene into genome to achieve long-term expression^27^,^28^, its application in non-invasively cell tracking and monitoring *in vivo* is thus limited. We have introduced SB transposon, which is highly efficient in gene integration^23^,^29^, to Bac-NIS system to overcome this obstacle.

In order to establish an efficient single plasmid expression system^30^,^31^, we designed an “all-in one” plasmid, in which a SB100X sequence and the NIS gene sequence is placed outside and between the two IR/DR sites to facilitate the “cut-and-paste” process of transposon. Briefly, the system works in following process: 1. specific binding of SBsase when it recognizes IR/DRs; 2. pairing of a synaptic complex within two ends as well as binding together by SBsase subunits; 3. excising from the donor locus; 4. recognizing the target sequence in genomes and reintegrating at the target locus^19^. The “cut-and-paste” mechanism of SB transposon mediated transgene integration into the genome has been proven by FISH assay^32^.

In current study, according to the results of flow cytometry, eGFP protein expression can last for 196 days with no obvious decline in G418 screened Bac-eGFP-SB-NeoR-U87 cells, which means the feasibility of achieving a long-term transgene expression by this novel hybrid system. Furthermore, a selective resistance gene is also a crucial part of the novel system for maximizing the advantage of application. The high positive proportion of NIS protein evaluated by cell immunofluorescence at the 196^th^ day as well as no obvious decline of the NIS mRNA and protein level at the 1^st^, 7^th^, 14^th^, and 28^th^ week in screened Bac-NIS-SB-NeoR-U87 cells, likewise further proved the capability of this novel hybrid system to sustain long-term transgene expression.

Besides, in a dynamic iodide uptake study, radioiodide rapidly increased intracellularly from 0 to 45 minutes, followed by a dynamic equilibrium during 45 to 120 minutes instead of an obvious decline after peaking compared to some studies^5^,^15^. The mechanism of dynamic uptake equilibrium consists with the equilibrium of intra/extra cellular ion change *in vitro* assay, because NIS function relies on the inwardly directed Na+ gradient^1^,^2^. Combined with the significant inhibition by perchlorate ion, the cloned NIS gene in the new hybrid system has a specific function of accumulating iodide, which is essential for the further application in cell tracking and monitoring *in vivo*. Furthermore, the iodide uptake assay at various weeks from the 1^st^ to 28^th^ week revealed the ability of accumulating iodine did not decline over time, which was consistent with the change of NIS mRNA and protein level.

*In vivo* SPECT imaging, a pinhole detector instead of parallel-hole detector was chosen due to the higher resolution. The transplanted Bac-NIS-SB-NeoR-U87 tumor cells were clearly imaged at 30 minutes in each imaging (20^th^, 28^th^, and 43^th^ day after transplantation), with a T/NT radio (5.30 ± 0.72, tumor/contralateral tissue). The control U87 cells, however, were not detected by SPECT imaging at any time point. Because of the metabolism in mice, the Bac-NIS-SB-NeoR-U87 cells imaging faded gradually with time in each imaging. In the SPECT images, the stomach and bladder were also visible because of the NIS protein existence in the stomach and ^131^I clearance from blood by kidneys. However, despite the NIS protein existence in the thyroid, the thyroid imaging was not as clear as the tumor, stomach, or bladder in the SPECT instrument. At 43 days, the Bac-NIS-SB-NeoR-U87 tumor was clearer than the first and second imaging at 30 minutes, due to the tumor growth accompanied by the increased NIS protein. The result of immunohistochemistry also revealed the NIS protein was abundant in the Bac-NIS-SB-NeoR-U87 tumor tissue, which was consistent with the specific tumor imaging in SPECT.

In our study, the novel hybrid Bac-NIS system (Bac-NIS-SB-NeoR) has been proven its capability to sustain long-term transgene expression both *in vitro* and *in vivo*. Considering the significant correlation between the radioactivity and cell number *in vitro* assay, this novel system also has a great potential value for non-invasively quantitatively monitoring transplanted stem cells or immune cells *in vivo*. Despite the insertion of transgene into the chromosome, the baculovirus-SB hybrid system is safer because of its near-random genomic insertion compared to the retrovirus, lentivirus and AAV-derived vector systems^33^, which revealed an integration bias into transcriptionally active units in the genome^34^,^35^,^36^,^37^.

Nevertheless, there is still a limitation in our novel vector because baculovirus can be rapidly inactivated by serum complement^38^, which may hinder the application of this novel hybrid system in genetic therapy by direct injection *in vivo*. Therefore, the new hybrid baculovirus-SB system, which can be used for mediating long-term reporter gene expression, may prefer in non-invasively monitoring in molecular imaging.

## Conclusion

In the present study, we have constructed a novel hybrid Bac-NIS-SB-NeoR system that can achieve a long-term NIS gene expression both *in vitro* and *vivo*, and is ideal for radionuclide reporter gene imaging, especially in cell tracking and monitoring *in vivo*. Considering the biosafety, low insertional mutagenesis risk, easy manipulation and production, and the long-term expression of transgene, this novel Bac-NIS-SB-NeoR system has great potential in nuclear molecular imaging in the future, especially in cell tracking and monitoring *in vivo*, as an alternative option for other virus reporter gene imaging systems.

## Methods

### Plasmids’ construction and recombinant baculovirus production

The CMV-eGFP with two sites of XbaI and EcoRI was amplified by polymerase chain reaction (PCR) from pFB-eGFP plasmid^5^ and cloned into pT2-BH (Addgene plasmid 26556) backbone to obtain the plasmid of pT2-BH-eGFP. After that, IR/DR-CMV-eGFP with AvrII and NotI sites amplified from pT2-BH-eGFP plasmid, CMV-SB100x with SalI and BstZ17I sites amplified from pCMV(CAT)-SB100x plasmid (Addgene plasmid 34879), pSV40-NeoR with EcoRI, and NheI sites amplified from pcDNA-GLP-1R plasmid (a gift from Shanghai Institute of Materia Medica), were cloned into pFast-Bac HTB vector(previously stored in our laboratory) with corresponding sites to obtain the hybrid plasmid vector of pFast-eGFP-SB-NeoR.

The NIS gene fragment amplified from the pFast-NIS plasmid^5^ was cloned into the backbone of pFast-eGFP-SB-NeoR plasmid at the sites of AscI and BspDI to obtain a new plasmid of pFast-NIS-SB-NeoR.

After plasmids were constructed, the production of two recombinant baculovirus carrying eGFP or NIS genes (Bac-eGFP-SB-NeoR and Bac-NIS-SB-NeoR) were prepared as described previously^5^. The end-point dilution^39^ and RT-PCR^40^ method were used for determining the titers of the two recombinant baculovirus.

### G418 screening infected U87 human glioma cells

U87 cells, which were cultured in DMEM medium (ThermoFisher Scientific, Shanghai, China) supplemented with 10% FBS (HyClone, Logan, UT) and 1% penicillin-streptomycin (ThermoFisher Scientific, Shanghai, China) in 37 °C incubator with 5% CO_2_, were used to test the ability of long-term transgene expression mediated by novel constructed hybrid system. The Bac-eGFP-SB-NeoR and Bac-NIS-SB-NeoR virus were added with final multiplicities of infection (MOI) of 0 and 200, respectively. After a four-hour infection, U87 cells were washed twice by PBS and replenished with fresh full medium. Forty-eight hours later, G418 (Sangon, Shanghai, China) at 200 ug/ml was used for screening infected U87 cells to obtain stable cell lines expressing eGFP or NIS gene (Bac-eGFP-SB-NeoR-U87 and Bac-NIS-SB-NeoR-U87).

### Flow cytometry and cell immunofluorescence determination

After G418 screening, the GFP^+^% in Bac-eGFP-SB-NeoR-U87 cells was observed by an inverted phase contrast fluorescence microscope and determined by a flow cytometry (BD Accuri C6, Mansfield, MA). Meanwhile, the GFP^+^% was also determined in the Bac-eGFP-ITR-U87 (original version), Bac-eGFP-SB-U87 (with no screening resistance gene) and Bac-eGFP-SB-NeoR-U87 (with no G418 screening). Bac-NIS-SB-NeoR-U87 cells were determined by cell immunofluorescence (anti-NIS mouse monoclonal antibody as primary antibody from Abcam, 1:200 and Alexa Fluor 488-conjugate anti-mouse IgG as second antibody from CST) after successfully screening.

### Dynamic iodide uptake of Bac- NIS -SB-NeoR-U87 cells

The modified study of dynamic iodide uptake was similar as previously performed^41^ except for the difference of a serum-free medium instead of HBSS. The stable Bac-NIS-SB-NeoR-U87 cells were seeded in a 24-well plate with a cell number of 5 × 10^4^. Twenty-four hours later, the seeded cells were replenished with 500 ul serum-fresh DMEM medium containing 3.7 kBq (0.1uCi) Na^125^I (Xinke, Shanghai, China) and 10 uM NaI (Sangon, Shanghai, China) per well. When uptake completed, each well was washed three times with cold PBS and incubated with 500 μl 1 M NaOH for 15 minutes. The radioiodide uptake was terminated and measured by a γ-counting machine at various time points of 0, 5, 15, 30, 45, 60, 75, 90 and 120 minutes.

### Inhibition of iodide uptake by ClO_4_^−^

Twenty-four hours before the inhibition experiments, the stable Bac-NIS-SB-NeoR-U87 cells were seeded in 24-wells with a density of 5 × 10^4^. Three concentrations (0, 50 and 500 uM) of NaClO_4_, together with 500 ul serum-free DMEM medium containing 3.7 kBq (0.1uCi) Na^125^I, and 10 uM NaI were added per well in each group, respectively. After 45 minutes of incubation in a 37 °C incubator with 5% CO_2_, the radioiodide uptake of each group was terminated and measured by a γ-counting machine.

### Correlation analysis between radioactivity uptake and cell numbers *in vitro*

The stable Bac-NIS-SB-NeoR-U87 cells were seeded at densities of 1 × 10^4^, 2 × 10^4^, 4 × 10^4^, 8 × 10^4^, and 1.6 × 10^5^ in a 24-well plate and incubated at 37 °C with 5% CO_2_ for 24 hours. Then 500 ul serum-free DMEM medium containing 3.7 kBq (0.1 uCi) Na^125^I and 10 uM NaI was added per well in each group. After 45 minutes incubation, the radioiodide uptake of each group was terminated and measured as above.

### Long-term iodide uptake measurements

At 1, 2, 3, 4, 6, 8, 10, 12, 16, 20, 24, and 28 weeks after successfully screening stable Bac-NIS-SB-NeoR-U87 cells, the radioiodide uptake was performed as above each week. 5 × 10^4^ cells were seeded in a 24-well plate and incubated at 37°C with 5% CO_2_ for 24 hours. Then radioiodide uptake terminated at 45 minutes was performed as above.

### Reverse-transcription polymerase chain reaction

Total RNA from stable Bac-NIS-SB-NeoR-U87 cells in different weeks was extracted using RNeasy Mini Kit (Qiagen, Hilden Germany) and transcribed into complementary DNA using PrimeScript RT Master Mix (Takara, Dalian, China) following the manufacturer’s instructions. SYBR Premix Ex Taq II (Takara, Dalian, China) was used for performing quantitative PCR (q-PCR) in a step-one, real-time PCR System (Applied Biosystems, Carlsbad, CA). Each threshold cycle (Ct) value was averaged from triplicate reactions, and relative NIS gene expression was calculated from the 2^−△△Ct^ value.

### Western blotting

Total protein from stable Bac-NIS-SB-NeoR-U87 cells in different weeks was extracted in a RIPA buffer(Beyotime, Shanghai, China) with a millesimal cocktail (Sangon, Shanghai, China). After protein quantification, equal protein was subjected to western blotting using mouse monoclonal anti-NIS antibody(NeoMarker, 1:500) and mouse monoclonal anti-GAPDH antibody(Sangon, 1:2000) as primary antibodies, followed by goat anti-mouse IgG-HRP(Beyotime, 1:2000) as a secondary antibody. Finally, chemiluminescence detection was used in the Rio-lab instrument.

### ^131^I SPECT imaging *in vivo*

All animal experiments were approved by the Ethics Committee of the Shanghai Jiao Tong University School of Medicine and were conducted in accordance with ethical principles governing animal welfare, rearing, and experimentation.

Male BALB/c nude mice that were 3–4 weeks of age (purchased from the Experimental Animal Centre of Ruijin Hospital, Shanghai, China) were subcutaneously injected in the armpit of the right upper limb with 100 μl of a suspension of Bac-NIS-SB-NeoR-U87 cells which had been cultured for approximately 28 weeks *in vitro*, followed by an equal amount of U87 cells in the left.

A hundred-microliter solution containing 500 μCi ^131^I was injected into the tail vein of each of 3 tumor-bearing mice at 20 days after the transplantation of tumor cells when the diameter of the tumors reached nearly 0.5 cm. A pinhole detector was used for acquiring an image in SPECT instrument(General Electric Company, WI, USA) at 30, 60, 90, 120, 180, and 240 minutes after the injection of ^131^I. Each time point image acquisition was under the same circumstances. The second and third *in vivo* imaging was performed with the same mice at 28 days and 43 days under the same conditions. The average radioactivity in tumor tissue at 30 minutes corresponding to three SPECT imaging was measured by the GE Xeleris image analysis system.

### Immunohistochemistry

At 43 days, nude mice were sacrificed by cervical vertebra dislocation, and tumor tissues were fixed in 4% paraformaldehyde for 48 hours, followed by paraffin embedding. Mouse monoclonal anti-NIS antibody(Abcam, ab17795, 1:500) was used at 4 °C overnight in immunohistochemistry, and images of each slice were acquired by a fluorescence microscope.

### Statistical analysis

All data are presented as the mean ± SD (at least N = 3). Statistical analysis was performed using Student’s t-test and One-way Analysis of Variance (ANOVA). A p value of less than 0.05 was considered statistically significant.

## Additional Information

**How to cite this article:** Lv, J. *et al*. A Novel Ideal Radionuclide Imaging System for Non-invasively Cell Monitoring built on Baculovirus Backbone by Introducing Sleeping Beauty Transposon. *Sci. Rep.* **7**, 43879; doi: 10.1038/srep43879 (2017).

**Publisher's note:** Springer Nature remains neutral with regard to jurisdictional claims in published maps and institutional affiliations.
